# Antimicrobial Stewardship: A Cross-Sectional Survey Assessing the Perceptions and Practices of Community Pharmacists in Ethiopia

**DOI:** 10.1155/2016/5686752

**Published:** 2016-11-22

**Authors:** Daniel Asfaw Erku

**Affiliations:** Department of Pharmaceutical Chemistry, School of Pharmacy, University of Gondar, Lideta kebele 16, P.O. Box 196, Gondar, Ethiopia

## Abstract

*Background*. Community pharmacists are key healthcare professionals for antimicrobial stewardship programs owing to their role in dispensing of antimicrobials. The aim of the present study was to assess the perception and practices of community pharmacists towards antimicrobial stewardship (AMS) in Ethiopia.* Methods*. A cross-sectional survey was conducted by selecting pharmacy sites through stratified simple random sampling technique. Descriptive and inferential statistics were used to analyze the data.* Results*. Majority of respondents strongly agreed or agreed that AMS program is vital for the improvement of patient care. Almost all of respondents agreed that pharmacists can play a prominent role in AMS and infection prevention (93.2%, median = 5; IQR = 2–5). However, only 26.5% of respondents strongly agreed or agreed that AMS should be practiced at community pharmacy level (median = 4, IQR = 1–3) and more than half of community pharmacists (59.9%) often/always dispense antimicrobial without a prescription.* Conclusion*. The present study revealed positive perceptions and practices of community pharmacists towards antimicrobial stewardship. Yet, some weak areas like integration of AMS program in community pharmacies, the significance of interprofessional involvement, and dispensing of antimicrobials without a valid prescription still need improvement.

## 1. Introduction

The emergence of antimicrobial resistance (AMR), which is thought to be the main cause of morbidity and mortality from otherwise treatable infections, is largely attributed to the use, overuse, or misuse of antimicrobials [1]. It is estimated that, every year, multidrug resistant (MDR) bacteria claim the lives of more than 20,000 people in USA, 25,000 patients in European union countries, and 90,000 people in southern Asia [2, 3]. In Ethiopia, there are signs of irrational use of antibiotics by patients as well as healthcare providers. According to the baseline survey conducted by Food, Medicine, and Healthcare Administration and Control Authority (FMHACA) of Ethiopia, about two-thirds of patients (70%) who visited outpatient clinics have had one or more antibiotics prescribed with a percentage of irrational prescribing close to 40% [4]. A number of researchers underlined the relationship between the inappropriate use of antimicrobials and the emergence of AMR [5].

AMR has a considerable impact on the healthcare system as it will result in a higher morbidity rate and prolonged duration of hospitalization [6]. Antimicrobial stewardship (AMS) is the practice of escalating and sustaining the rational use of antimicrobials to optimize patient outcomes, reduce costs, and avoid the collateral side effects linked with these medications [7]. Taking into consideration the rising AMR worldwide, numerous researchers have encouraged healthcare providers to hold the responsibility of AMS in practice areas. Community pharmacists are often regarded as key healthcare providers for AMS programs owing to their role in dispensing of antimicrobials [7]. They also bring their exclusive knowledge of pharmacokinetic, pharmacodynamic, and pharmacoeconomic principles to antimicrobial therapy, which ultimately improves patients' health outcomes [8, 9]. Taking into account the potential role of community pharmacists in the development and execution of AMS programs, it is imperative to know the perceptions and practices of community pharmacists towards AMS. Few studies regarding the practice and role of community pharmacists in AMS were done elsewhere [10] in the globe but there is a scarcity of data in Ethiopia. The present study was conducted to assess the perception and practices of community pharmacists towards AMS.

## 2. Materials and Methods 

### 2.1. Study Design and Sampling

A descriptive cross-sectional survey was conducted among Ethiopian community pharmacists to assess their perceptions and practices towards AMS. All community pharmacists practicing within pharmacy profession in Ethiopia were approached. Stratified simple random sampling technique was applied and stratified into historical and nonhistorical advantaged regions. In Ethiopia, there are nine regions (Tigray, Afar, Amhara, Oromia, Somalia, Harari, Benishangul, People of southern nation, and nationalities as well as Gambela) and two city administrations (Dire Dawa and Addis Ababa). Among these, the study was conducted in Dire Dawa and Addis Ababa, historical advantaged cities (Gondar, Jimma, and Mekelle), and Adama, Hawassa, and Dessie (nonhistorical regions). Single proportion formula was applied to draw the sample size [11]. The Federal Ministry of Health- (FMOH-) Health Sector Development Program (HSDP) IV (2010–2015) was used to draw the sample size. According to FMOH-HSDP IV, a total of 661 community pharmacists were present by the end of 2010 in the country [12]. Accordingly, 449 participants were selected based on proportion to size and questionnaires were equally distributed in all the eight cities.

### 2.2. Data Collection and Management

A validated self-administered questionnaire developed by Khan et al. [10] was used to collect the data. The questionnaire was first prepared in English and translated to local language (Amharic) and then backtranslated to English to make sure the translated version gives the proper meaning. The content validity of the modified data collection tool was confirmed by a team of experts including a senior physician with infectious disease expertise, a public health personnel, and clinical pharmacist and it was pretested on 20 community pharmacists prior to the gross data collection, which were excluded from final analysis, and appropriate modifications were instituted. The final data collection tool included 5 questions to assess the sociodemographic characteristics of respondents, 8 questions to assess the perceptions of respondents towards AMS, and 10 questions to assess the practice of respondents towards AMS. The responses on perception were based on a Likert scale of 1 to 5 (1: strongly disagree, 2: disagree, 3: neutral, 4: agree, and 5: strongly agree). For practice questions, scores were given as 1, never; 2, rarely; 3, occasionally; 4, often; and 5, always. The negative statements were reverse scored during the analysis so that higher scores reflect a positive perception and practice towards AMS.

### 2.3. Data Analysis

The data collected were entered into and analyzed using statistical package for social sciences (SPSS) software version 21.0 for Windows. Frequencies, percentages, median, and interquartile range (IQR) were computed. Mann–Whitney* U* and Kruskal-Wallis test were applied to detect the differences in median scores. Shapiro-Wilks and Kolmogorov-Smirnov tests were employed to test the normality of the data. Bonferroni adjustment was also utilized to examine the significance of intergroup variables and a *p* value of less than 0.05 was considered as statistically significant.

### 2.4. Ethical Considerations

The study was approved by the ethical review committee of the School of Pharmacy, University of Gondar. Informed consents from each city's administrators and all participant pharmacists were also gained prior to conducting the study. The data collected were kept anonymous.

## 3. Results

Among 449 community pharmacists approached, 389 completed the survey giving a response rate of 86.6%. Among the respondents, 244 (62.7%) were males, with a mean (with SD) age of 29.8 ± 7.6 years. The sociodemographic characteristics of respondents are shown in Table 1. The median with interquartile ranges of the perception and practice scores of respondents towards AMS are presented in Tables 2 and 3.

Majority of respondents agreed that AMS program is vital for the improvement of patient care (86.3%, median = 5; IQR = 2–5) and that pharmacists can play a prominent role in AMS and infection prevention (93.2%, median = 5; IQR = 2–5). Likewise, majority of respondents agreed that pharmacists should attend different conferences, seminars, and workshops about AMS for a better understanding and practice (90.5%, median = 5, IQR = 2–5). Similarly, more than two-thirds of respondents did not agree on the statement that prescribing physicians are the only healthcare providers who need to understand AMS (69.7%, median = 4, IQR = 2–4). In contrast, only 26.5% of respondents agreed that AMS should be practiced at community pharmacy level (median = 4, IQR = 1–3).

Self-reported practices of community pharmacists towards AMS were also assessed. Accordingly, majority of respondents always/often communicate with prescribers in case of ambiguity about the correctness of antibiotic prescription (77.9%, median = 4, IQR = 1–4). However, more than half of community pharmacists (59.9%) often/always dispense antimicrobial without a valid prescription. Similarly, more than half of respondents often/always dispense antibiotics for a duration longer than what is prescribed by the physicians (59.4%, median = 4, IQR = 2–4). Collaboration with other healthcare providers on antimicrobial use was often done by 54% of respondents (median = 4, IQR = 2–4). It was also observed that only 31.1% respondents often participate in antimicrobial awareness movements (median = 4, IQR = 2–4). Respondents with undergraduate degree had a significantly lower median scores with respect to their perceptions (median score: 3 versus 4, *p* value 0.020) and practice about AMS (median score: 3 versus 4, *p* value 0.012) than respondents with postgraduate degree. Similarly, respondents with less than five years of experience had considerably lower median scores with respect to their perceptions (median scores: 3.5 versus 4, *p* value 0.031) and practice (median scores: 3 versus 4, *p* value 0.015) than participants with greater than five years of experience (Table 4).

## 4. Discussion

In many sub-Saharan African countries like Ethiopia, infectious and communicable diseases accounted for the majority of morbidities and mortalities [13]. The frequent and injudicious use of antimicrobials coupled with the unlimited accessibility has led to a greater level of antimicrobial resistance [14]. Due to this, numerous researchers have encouraged the healthcare professionals to hold the responsibility of AMS in practice areas. Pharmacists are recognized for having an integral role in interprofessional AMS activities [8]. To the best of our knowledge, this study is the first to investigate the perceptions and practices of community pharmacists towards AMS in Ethiopia.

The results of the present study revealed positive perceptions and practices of community pharmacists towards AMS. The findings of this study strengthen the preceding work indicating the growing role of pharmacists in curbing the injudicious use of antibiotics [15, 16]. Majority of community pharmacists agreed that AMS program is vital for the improvement of patient care. A study done in Malaysia supports the finding of our study that more than 90% of pharmacists agreed on the importance of AMS for optimal patient care [10]. There is also appealing evidence supporting the notion that AMS is vital for optimal patient care [17]. Majority of respondents also agreed that community pharmacists have a prominent role in AMS program in healthcare settings which is in line with studies done elsewhere [7, 10]. However, it is worth mentioning that most of pharmacists were neutral to the statement about the integration of AMS programs in community pharmacy settings. This could be due to the underutilization of community pharmacists in combating the problem of antimicrobial resistance, even though evidences support that community pharmacist could take a prominent role in rational use of antibiotics [10, 18]. In Ethiopia, there is no recognized AMS program executed in community pharmacies. By taking advantage of the mostly optimistic views of community pharmacists on their role in AMS programs, the Federal Ministry of Health, hospital administrations, and other stakeholders should provide strategies on how they could take action as AMS proponents. A number of researchers put forward the idea that interprofessional communication is vital for AMS program to be effective [19]. The findings of the present study corroborate this notion as a greater number of community pharmacists communicate with the prescriber in case of ambiguity about the correctness of antibiotic prescription. Similarly, the study done in Malaysia [10] reported a similar finding.

Apart from monitoring the antibiotic prescribing practice, continuous supervision of antibiotic dispensing practice is a key approach to hold antibiotic resistance. This notion is further supported by the study done in Australia which reported that improving antimicrobial prescribing and dispensing would reduce the emergence of antimicrobial resistance [20]. The findings of our study indicate that a significant number of community pharmacists dispense antibiotics without a valid prescription. In a recent simulated patient study conducted by Erku et al., it was reported that a substantial number of community pharmacies in Addis Ababa, Ethiopia, dispensed antibiotics without a valid prescription and most of the medications were dispensed inappropriately [21]. A substantial proportion of the total drug budget in many developing countries, including Ethiopia, is dedicated to antimicrobials and they are often the largest group of drugs purchased [22]. Even though the extent of nonprescription sale of antibiotics is not extensively studied in Ethiopia, the financial incentives and business orientation of pharmacies, which are known to be reasons for dispensing malpractice [23], need to be fully resolved for planning intervention strategies to curb antimicrobial resistance. The present study showed that about 42.1% of community pharmacists often/always took part in antimicrobial awareness movements. Our finding corroborates with a study done in Australia which showed that community pharmacists were more eager to join the AMS movements in healthcare settings [20]. In contrast, a study done in Malaysia demonstrated that greater part of pharmacists rarely/occasionally takes part in such campaigns [10].

## 5. Limitation

The present study highlights an area of pharmacy practice where there is scarcity of literature. However, the present study has some limitations that should be considered while interpreting the results. The study was a cross-sectional survey conducted in 8 cities and the results cannot be generalized to other cities in Ethiopia. The high response rate reported in this study could be due to the fact that some of the respondents may provide extreme responses as compared to others and might be subjected to recall bias as the self-administered questionnaire which depends on honesty and faith of the respondents. Furthermore, it would have been more helpful to explore for which conditions antibiotics were dispensed and a disease-specific approach was taken. Despite the above limitations, our findings have significant implications for improving the use of antibiotics in community settings.

## 6. Conclusions

The present study revealed positive perceptions and practices of community pharmacists towards AMS. Yet, some weak areas like integration of AMS program in community pharmacies, the significance of interprofessional involvement, and dispensing of antimicrobials without a valid prescription still need improvement. Interventions to further improve the perception and practices of community pharmacists towards AMS must be tailored to target the gaps underlined in this study. Further large scale studies are necessary to validate the findings of the present study by including a greater number of community pharmacists in Ethiopia.

## Figures and Tables

**Table 1:** Demographic characteristics of community pharmacists in Ethiopia (*N* = 389).

Characteristics	Frequency (%)
Mean age (years)	29.8 ± 7.6 (SD)
*Gender*	
Male	244 (62.7)
Female	145 (37.3)
*Highest pharmacy degree achieved*	
Diploma	64 (16.5)
Bachelors (B.Pharm)	280 (72)
Postgraduation (MSc.)	45 (11.6)
*Work experience*	
<5 years	219 (56.3)
>5 years	170 (43.7)
*Type of pharmacy *	
Independent pharmacy	139 (35.7)
Drug store	128 (32.9)
Chain pharmacy	122 (31.4)

SD: standard deviation.

**Table 2:** Community pharmacists' perception towards AMS (*N* = 389).

Statements	Strongly disagree	Disagree	Neutral	Agree	Strongly agree	Median (IQR)
AMS improves patient care	0	0	53 (13.6%)	123 (31.6%)	213 (54.7%)	5 (2–5)
AMS should be practiced at community pharmacy level	67 (17.2%)	39 (10%)	180 (46.3%)	33 (8.5%)	70 (18%)	4 (1–3)
AMS reduces problem of antimicrobial resistance	0	23 (5.9%)	45 (11.6%)	102 (26.2%)	219 (56.3%)	5 (3–5)
Sufficient education on AMS should be given to community pharmacists	30 (7.7%)	35 (8.9%)	89 (22.9%)	143 (36.8%)	92 (23.6%)	3 (2–4)
Community pharmacists should attend relevant conferences and workshops on AMS for better understanding and practice	0	9 (2.3%)	28 (7.2%)	72 (18.5%)	280 (72%)	5 (2–5)
Individual efforts at antimicrobial stewardship have negligible impact on antimicrobial resistance problem	80 (20.6%)	43 (10%)	110 (28.3%)	39 (10%)	117 (30.1%)	3 (2–4)
Doctors are the only healthcare professionals who need to understand AMS	159 (40.9%)	112 (28.8%)	51 (13.1%)	50 (12.8%)	17 (4.4%)	4 (2–4)
Community pharmacists have a responsibility to take a prominent role in AMS and infection prevention	0	0	30 (7.7%)	109 (28.9%)	250 (64.3%)	4 (2–5)

**Table 3:** Community pharmacists' practice towards AMS (*N* = 389).

Statements	Never	Rarely	Occasionally	Often	Always	Median (IQR)
I dispense antimicrobial on prescription with complete clinical information	78 (20%)	99 (25.4%)	145 (37.3%)	39 (10%)	28 (7.2%)	4 (2–4)
I dispense antimicrobials without a prescription	38 (9.8%)	97 (24.9%)	21 (5.4%)	144 (37%)	89 (22.9%)	4 (2–4)
I dispense antimicrobial for more than the prescribed duration on patient's request	34 (8.7%)	51 (13.1%)	73 (18.8%)	154 (39.6%)	77 (19.8%)	4 (2–4)
I evaluate the antimicrobial prescription in accordance with good dispensing practice guideline	75 (19.3%)	89 (22.9%)	95 (24.4%)	36 (9.2%)	94 (24.2%)	4 (2–4)
I collaborate with other healthcare professionals for AMS and infection prevention	11 (2.8%)	21 (5.4%)	112 (28.8%)	210 (54%)	35 (9%)	4 (2–4)
I communicate with prescribers if I am unsure about the appropriateness of an antibiotic prescription	8 (2%)	38 (9.8%)	40 (10.3%)	160 (41.1%)	143 (36.8%)	4 (1–4)
I consider clinical and safety parameters like drug interaction, ADRs, and allergy before dispensing the antibiotic prescribed	21 (5.4%)	23 (5.9%)	70 (18%)	188 (48.3%)	87 (22.4%)	4 (2–4)
I take part in antimicrobial awareness movements to promote the rational use of antimicrobials	80 (20.6%)	43 (11%)	102 (26.2%)	121 (31.1%)	43 (11%)	4 (2–4)
I educate patients on the use of antimicrobials and drug resistance issues	21 (5.4%)	25 (11.6%)	175 (45%)	74 (19%)	94 (24.2%)	4 (3–4)
I make efforts to prevent or reduce the transmission of infections within the community	38 (9.8%)	45 (11.6%)	112 (28.8%)	127 (36.6%)	67 (17.2%)	4 (2–4)

**Table 4:** Median perception and practice scores of participants regarding AMS (*N* = 389).

Variable	Perception (median score)	Rank	*p* value	Practice (median score)	Rank	*p* value
*Gender *						
Male	4	97.5	0.584	4	89.4	0.089
Female	4	94.4		4	94.2	
*Age, in years*						
20–30	4	88.6	0.300	4	96.8	0.675
31–40	4	92.6		4	95.3	
41–50	4	81.8		4	89.7	
*Qualification *						
Undergraduate	3	97.3	0.020^*∗*^	3	83.8	0.012^*∗*^
Postgraduate	4	142.8		4	132.2	
*Experience *						
Less than 5 years	3.5	79.4	0.031^*∗*^	3	80.7	0.015^*∗*^
Greater than 5 years	4	98.1		4	107.3	

^*∗*^Statistically significant association (*p* < 0.05).
